# Reduction of in-hospital non-COVID-19 pneumonia in stroke patients during the COVID-19 pandemic

**DOI:** 10.1007/s10072-023-06712-0

**Published:** 2023-02-28

**Authors:** Raffaele Ornello, Enrico Colangeli, Giulia Ceccanti, Leondino Mammarella, Giovambattista Desideri, Simona Sacco

**Affiliations:** 1 Department of Biotechnological and Applied Clinical Sciences, University of L’Aquila, Via Vetoio 1 Coppito, 67100 L’Aquila, Italy; 2 Department of Emergency Medicine, Ospedale “Felice Lotti”, Pontedera, Italy; 3 Servizio Flussi Informativi E Statistica Sanitaria, ASL 1 Abruzzo, Avezzano, L’Aquila Italy; 4 Department of Internal Medicine, Public Health, Life and Environmental Sciences, University of L’Aquila, L’Aquila, Italy

**Keywords:** Stroke, Infections, Pneumonia, Urinary tract infections, COVID-19

## Abstract

**Background:**

Measures adopted to contain the spread of SARS-CoV-2 could have led to a reduction in the rate of non-COVID-19 infections. We assessed whether a similar reduction was present in patients with stroke.

**Methods:**

We performed a hospital-based study nested in a prospective population-based registry. We compared prevalence of infections and in-hospital mortality in subjects admitted for acute stroke between the first pandemic year (study period, from March 2020 to February 2021) and the pre-pandemic year (control period, from March 2019 to February 2020). Infections were reported as pneumonia (PNA), urinary tract infections (UTI), and any infection (INF).

**Results:**

From the control (*n* = 677) to the study period (*n* = 520), the prevalence of INF decreased from 11.5 to 4.6% (*p* < 0.001) and that of PNA decreased from 6.9 to 2.5% (*p* = 0.001). No changes in in-hospital mortality and length of hospital stay were observed between the two periods.

**Conclusions:**

The observed reduction of in-hospital pneumonias in patients with stroke was likely attributable to the use of protective measures and limitation of hospital visits. Maintaining some of those measures in the long term may contribute to control infections in hospitalized patients with stroke.

## Introduction

The COVID-19 pandemic had a profound impact on care for non-communicable diseases [1] including stroke [2, 3]. While overall health systems were adversely affected by the pandemic, the adoption of stringent measures to prevent the spread of SARS-CoV-2 may have provided benefits in control of other infections. This is suggested by some reports [4, 5] but has not been explored in the setting of dedicated acute stroke care. We assessed whether the COVID-19 pandemic period was associated with a reduction of in-hospital infections in patients hospitalized with stroke.

## Methods

Our study complies with the Strengthening The Reporting of OBservational studies in Epidemiology (STROBE) statement [6] (Supplementary Material).

This hospital-based study is nested within an ongoing prospective population-based registry conducted in the 298,343 residents [7] in the district of L’Aquila, central Italy. The district hosts 4 public hospitals, two of which have Stroke Units. The protocol was approved by the Internal Review Board of the University of L’Aquila with registration number 13/2018. Informed consent was obtained from the study participants or their authorized proxies. Data supporting findings of the present study are available from the corresponding author upon reasonable request.

We considered the study period as the first pandemic year from March 1, 2020, to February 28, 2021; comparisons were made with the period spanning from March 1, 2019, to February 29, 2020. The system of care for stroke did not change during the COVID-19 outbreak in the district. However, hospital protocols included mandatory use of surgical masks for all hospital staff, frequent use of sanitizing gel for hand hygiene, and visitor access restrictions; visitors also had to wear face masks and use sanitizing gel before entering wards.

We included all patients hospitalized for any type of stroke (cerebral ischemia, intracerebral hemorrhage, subarachnoid hemorrhage) within the study period. Two neurologists (RO and SS) validated the diagnoses. The presence of infections was assessed by checking medical records for pneumonia (PNA, ICD-9-CM codes 482–486), urinary tract infections (UTI, ICD-9-CM code 599), and generic infections without specified site (ICD-9-CM code 041). Diagnoses were validated by a geriatrician (GC). We excluded patients with COVID-19 including those with pneumonia.

Recorded information included patients’ age, sex, stroke type (ischemic stroke, intracerebral or subarachnoid hemorrhage), type of infection, Stroke Unit admission, length of hospital stay, and in-hospital mortality. Infections were reported as PNA, UTI, and any infection (INF, as a cumulative outcome of PNA and/or UTI and/or other generic infections without specified site).

### Statistical analysis

We compared the yearly and quarterly (March–May, June–August, September–November, December–February) rates of PNA, UTI, and INF in the study period compared with the control period. Comparisons were made by the chi-squared test or Fisher exact test as appropriate. The distribution of patients’ characteristics between the study and control periods, including sex, median age, stroke subtypes, Stroke Unit admission, median length of hospital stay, and in-hospital mortality, was compared by the chi-squared or Wilcoxon test as appropriate, to verify comparability. Analyses were performed with the R software, version 4.1.2.

## Results

**Fig. 1:**
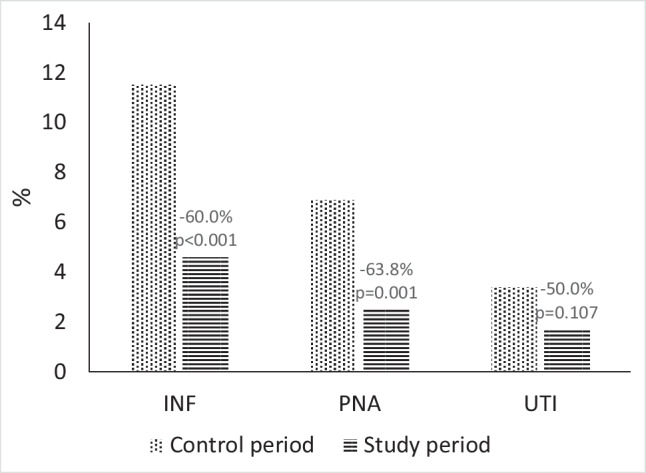
Rates of any infection (INF), pneumonia (PNA), and urinary tract infections (UTI) in patients with stroke during the study period (March 2020–February 2021) and the control period (March 2019–February 2020). Percentages on column show percent changes from the control to the study period

**Fig. 2:**
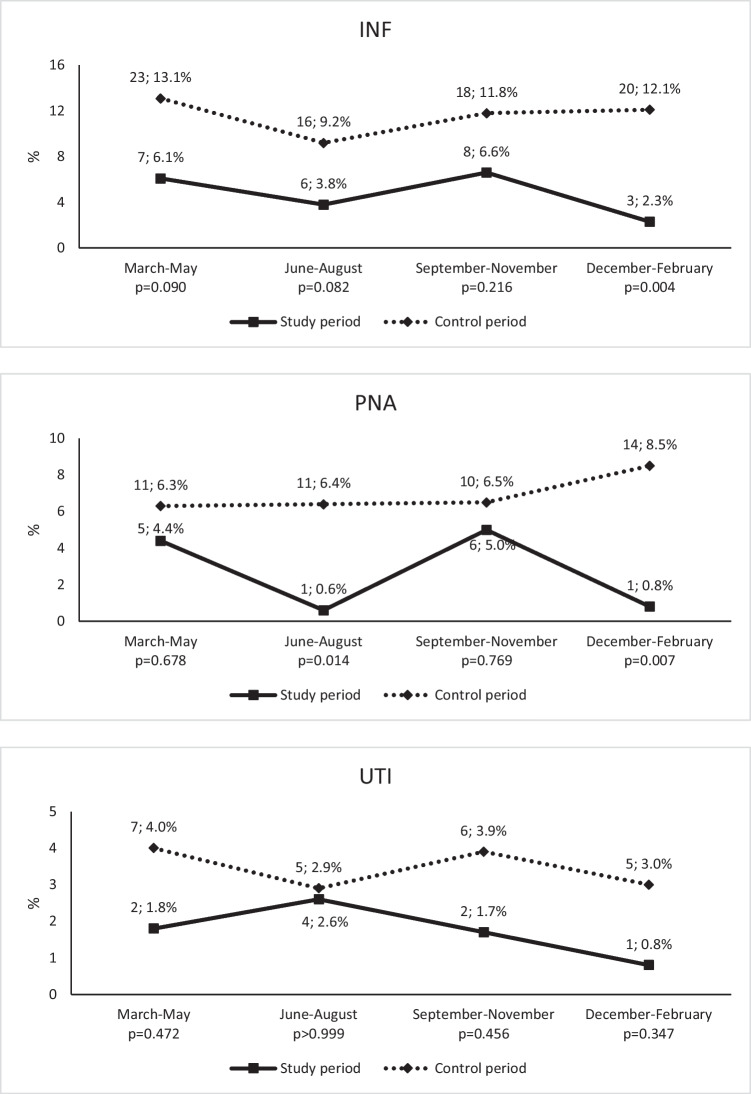
Quarterly rates of any infection (INF), pneumonia (PNA), and urinary tract infections (UTI) in patients with stroke during the study and control periods

Patients hospitalized with stroke decreased from 667 in the control period to 520 in the study period (− 23.0%; *p* < 0.001). Patients’ characteristics did not differ between the two periods, except from a lower proportion of patients with stroke admitted to a Stroke Unit from the control to the study period (61.4 to 49.6%, *p* < 0.001; Table 1). From the control to the study period, INF decreased from 77 (11.5%) to 24 (4.6%; *p* < 0.001); in detail, PNA decreased from 46 (6.9%) to 13 (2.5%; *p* = 0.001), while UTI changed from 23 (3.4%) to 9 (1.7%; *p* = 0.103; Fig. 1). The decreases in the rate of PNA were significant during the second (June–August) and fourth quarter (December–February) of the considered years (Fig. 2). There was no difference in median length of hospital stay (7 vs 6 days, *p* = 0.826) and in-hospital case-fatality (11.3% vs 11.5%, *p* = 0.989) between the study and the control periods (Table 1).

**Table 1:** Comparison between populations in the study and control periods. IQR indicates interquartile range

	Study period (*n* = 520)	Control period (*n* = 667)	*p* value
Male, *n* (%)	276 (53.1)	335 (50.2)	0.359
Age, median (IQR)	76 (66–84)	78 (66–85)	0.823
Stroke type, *n* (%)			0.857
Ischemic stroke	398 (76.5)	518 (77.7)	
Intracerebral hemorrhage	93 (17.9)	116 (17.4)	
Subarachnoid hemorrhage	29 (5.6)	33 (4.9)	
Quarter, *n* (%)			0.247
I (March–May)	114 (21.9)	176 (26.4)	
II (June–August)	156 (30.0)	173 (25.9)	
III (September–November)	121 (23.3)	153 (22.9)	
IV (December–February)	129 (24.8)	165 (24.7)	
Stroke Unit admission, *n* (%)	256 (49.6)	407 (61.4)	< 0.001
Days of hospital stay, median (IQR)	7 (4–11)	6 (4–11)	0.826
In-hospital death, *n* (%)	59 (11.3)	77 (11.5)	0.989

## Discussion

We observed a decrease in infections in patients hospitalized for stroke during the COVID-19 pandemic. In detail, the decrease was evident for PNAs, but not UTIs, which suggests a specific impact of preventive measures for airborne infections that can be attributed to the use of face masks, restriction of visits, and enhanced sanitization. Previous reports of decreased incidence of non-COVID-19 infections during the COVID-19 pandemic [4, 5] were not focused on patients with stroke. In our study, reduction of infections was not paralleled by reduced mortality or length of hospital stay possibly because of the low numbers of patients with infections in this analysis. Nevertheless, it is well known that infections—and mostly PNA [8]—can prolong hospital stay and increase mortality [9]; consequently, their prevention represents an important contribution to better stroke outcomes.

Considering our results, it may be suggested that maintaining use of face masks, restriction in visits to hospitalized patients, and enhanced sanitization in the long term could provide a benefit to patients with stroke. However, continuous use of face masks by the hospital staff could lead to discomfort, problems with communication, adherence, and misuse in the long term [10]. We have also to consider the additional cost and hospital waste brought by continuous use of face masks [11]. Besides, limitation of visits to patients might be associated with psychological distress and feelings of loneliness by patients, thus increasing the risk of delirium [12]. Lack of assistance by caregivers might also increase the burden on the hospital staff.

The strengths of our study include linkage to a population-based registry, comparable populations between the study and control periods, and additional analyses according to infection type and seasonality. As stated above, a limitation of the study is the low number of included patients with infections both before and during the pandemic, which could explain the lack of difference in in-hospital mortality between the two periods. Additionally, the diagnosis of infections was assessed by checking the codes in the medical records and we could not distinguish community-acquired from hospital-acquired pneumonia. We also cannot exclude that the decreased proportion of patients with stroke admitted to a Stroke Unit and the lowered attention to non-COVID-19 infections during the pandemic could have led to an underdiagnosis of infections. We do not have clear explanations for decreased admissions to Stroke Units; however, they could be determined at least in part by the reorganization of acute health care during the pandemic. We did not retrieve data about the severity of infections, as well as their long-term consequences. It is also worth noting that post-stroke pneumonia is not necessarily airborne but may be caused by weakness of swallowing leading to aspiration [13, 14]. Besides, we cannot fully exclude that other factors, such as decreased hospitalizations for stroke [2, 3], could have had a role in the decrease of non-COVID-19 infections in hospital settings.

In conclusion, during the COVID-19 pandemic, we observed a reduction of in-hospital pneumonias in patients with stroke, likely attributable to measures to contain COVID-19. Maintaining some of those measures in the long term may help controlling infections in patients with stroke and potentially improve patients’ outcomes.

## Data Availability

The datasets generated during and/or analyzed during the current study are available from the corresponding author on reasonable request.

## References

[1] Sheldon TA, Wright J (2020) Twin epidemics of covid-19 and non-communicable disease. BMJ m2618. 10.1136/bmj.m2618 10.1136/bmj.m2618 32605906

[2] Sacco S, Ricci S, Ornello R (2020). Reduced admissions for cerebrovascular events during COVID-19 outbreak in Italy. Stroke.

[3] Nogueira RG, Abdalkader M, Qureshi MM (2021). Global impact of COVID-19 on stroke care. Int J Stroke.

[4] Huang C (2021) The COVID-19 pandemic and the incidence of the non-COVID-19 pneumonia in adults. Front Med (Lausanne) 8: 10.3389/fmed.2021.737999 10.3389/fmed.2021.737999 PMC8632034 34859006

[5] Tanislav C, Kostev K (2022). Fewer non-COVID-19 respiratory tract infections and gastrointestinal infections during the COVID-19 pandemic. J Med Virol.

[6] von Elm E, Altman DG, Egger M (2007). The Strengthening the Reporting of Observational Studies in Epidemiology (STROBE) statement: guidelines for reporting observational studies. The Lancet.

[7] Istituto Nazionale di Statistica (ISTAT) (2011) Censimento della popolazione e delle abitazioni 2011. https://dati.istat.it. Accessed 25 July 2022

[8] Katzan IL, Cebul RD, Husak SH (2003). The effect of pneumonia on mortality among patients hospitalized for acute stroke. Neurology.

[9] Westendorp WF, Nederkoorn PJ, Vermeij J-D (2011). Post-stroke infection: a systematic review and meta-analysis. BMC Neurol.

[10] Bakhit M, Krzyzaniak N, Scott AM (2021). Downsides of face masks and possible mitigation strategies: a systematic review and meta-analysis. BMJ Open.

[11] Feng S, Shen C, Xia N (2020). Rational use of face masks in the COVID-19 pandemic. Lancet Respir Med.

[12] Manca R, de Marco M, Venneri A (2020) The impact of COVID-19 infection and enforced prolonged social isolation on neuropsychiatric symptoms in older adults with and without dementia: a review. Front Psychiatry 11: 10.3389/fpsyt.2020.585540 10.3389/fpsyt.2020.585540 PMC7649825 33192732

[13] Kumar S, Selim MH, Caplan LR (2010). Medical complications after stroke. Lancet Neurol.

[14] Johnston KC, Li JY, Lyden PD (1998). Medical and neurological complications of ischemic stroke. Stroke.

